# Is San Diego California on Track to Reach HCV Elimination? A Modeling Analysis of Combination Prevention Strategies

**DOI:** 10.3390/v16121819

**Published:** 2024-11-22

**Authors:** Jaskaran S. Cheema, Scott Suckow, Christian Ramers, Patrick Loose, Andrea Tomada, Samantha Tweeten, Tara Stamos-Buesig, Daniela Abramovitz, William H. Eger, Steffanie A. Strathdee, Natasha K. Martin

**Affiliations:** 1 Division of Infectious Diseases and Global Public Health, University of California San Diego, La Jolla, CA 92093, USA; jacheema@health.ucsd.edu (J.S.C.); christianr@fhcsd.org (C.R.); dabramovitz@health.ucsd.edu (D.A.); wheger@health.ucsd.edu (W.H.E.); sstrathdee@health.ucsd.edu (S.A.S.); 2 The Liver Coalition, San Diego, CA 92121, USA; scott@livercoalition.org; 3 Family Health Centers San Diego, San Diego, CA 92123, USA; 4 County of San Diego Health and Human Services Agency, San Diego, CA 92123, USA; patrick.loose@sdcounty.ca.gov (P.L.); andrear.tomada@sdcounty.ca.gov (A.T.); samantha.tweeten@sdcounty.ca.gov (S.T.); 5 Harm Reduction Coalition San Diego, San Diego, CA 92101, USA; harmreduxsd@gmail.com

**Keywords:** people who inject drugs, elimination, hepatitis c, testing, harm reduction

## Abstract

In 2020, the *Eliminate Hepatitis C Initiative* in the county of San Diego (COSD) was launched, a private–public joint endeavor between the COSD and the American Liver Foundation. We use epidemic modeling to assess whether the COSD is on track to reach its elimination targets (80% reduction in incidence, 65% reduction in hepatitis C virus (HCV)-related mortality by 2030 compared to 2015) and what intervention scale-up may be required. We adapted a previously developed dynamic, deterministic model of HCV transmission and disease progression among adults in the COSD, stratified by risk, age, gender, and human immunodeficiency virus (HIV) status. The model is calibrated to detailed historical epidemiological data on HCV burden, treatment, and mortality in the COSD. We project HCV infections and mortality under status quo HCV treatment (65%/year among people coinfected with HCV and HIV, 0–5%/year among others) and determine what treatment scale-up among those without HIV is required to achieve HCV elimination, with or without concomitant reductions in injection transmission risk from 2024 onward. We project an increase in new HCV infections in the COSD to 2213 [95% C.I.: 1069–3763] in 2030, a mean 91% relative increase between 2015 and 2030. HCV-related deaths are expected to decrease to 246 [95% C.I.: 180–295] in 2030, a mean relative decrease of 14% compared to 2015. The incidence elimination target could be achieved through increasing HCV treatment among those without HIV to a mean of 60%/year, similar to the level achieved among people coinfected with HCV and HIV. Combination interventions reduce the treatment needed; if injecting risk is reduced by 25%, then treating 48%/year could achieve elimination. The COSD is likely not on track to reach the incidence or mortality targets, but achieving the incidence target is possible if treatment rates overall are scaled-up to rates that have been achieved among people coinfected with HCV and HIV. Elimination is achievable but requires committed funding and expansion of comprehensive testing, linkage, and treatment programs alongside harm reduction initiatives.

## 1. Introduction

Hepatitis C is a liver infection caused by the hepatitis C virus (HCV), which if left untreated can result in cirrhosis, liver failure, and death [1]. HCV transmission in the United States most commonly occurs due to used or unsterile syringe sharing among people who inject drugs, but vertical transmission and sexual transmission (particularly among men who have sex with men, MSM) can also occur, although less frequently [1]. Acute infection is often asymptomatic, with approximately one-quarter of individuals self-clearing their HCV infection and the remainder progressing to a chronic infection. Those with chronic infection are often asymptomatic until they develop some form of advanced liver disease, such as cirrhosis [1]. There is currently no effective vaccine for HCV; however, highly effective, all-oral direct-acting antiviral treatments now exist that can cure infection in >95% of individuals [1].

In 2015, the World Health Organization (WHO) set strategic goals to eliminate HCV as a public health threat [2], including an 80% reduction in HCV incidence and 65% reduction in HCV mortality by 2030 compared to the 2015 baseline [2]. Subsequently, many countries and local jurisdictions have developed action plans to achieve these HCV elimination targets. In 2023, the White House proposed an HCV elimination plan for the United States, requesting USD 11 billion over five years to support testing, treatment, and education [3]. However this initiative is in Congress where it has not yet received approval [4].

The county of San Diego (COSD) in California has an estimated 55,354 individuals with a history of HCV [5], and several associated HCV elimination initiatives. In 2018, the University of California San Diego (UCSD) Owen Clinic, a large HIV provider in the COSD, launched a micro-elimination initiative to scale-up HCV treatment among people coinfected with HCV and HIV. In 2020, the *Eliminate Hepatitis C Initiative* in the COSD was launched, a private–public joint endeavor between the COSD and the American Liver Foundation to support the achievement of the WHO goals [6]. This resulted in an implementation plan for the initiative, released in 2021, with the aim of promoting awareness of HCV and implementing prevention, screening, linkage, and treatment for HCV to reach the WHO incidence and mortality targets. Although our foundational work established a baseline for HCV burden in the COSD, and recent progress has been made in scaling up treatment among people coinfected with HCV and HIV, it is unknown what level of intervention scale-up is required to achieve the elimination targets in the COSD. Simulation modeling has been a tool for assessing the level of intervention scale-up required to achieve the HCV elimination targets and whether locations are on track to achieve elimination [7].

Using dynamic epidemic modeling of HCV transmission and disease progression, we aim to assess whether the COSD is on track to achieve its HCV elimination targets and, if not, what level of intervention scale-up will be required to meet them by 2030.

## 2. Methods

### 2.1. Model Description

**Figure 1:**
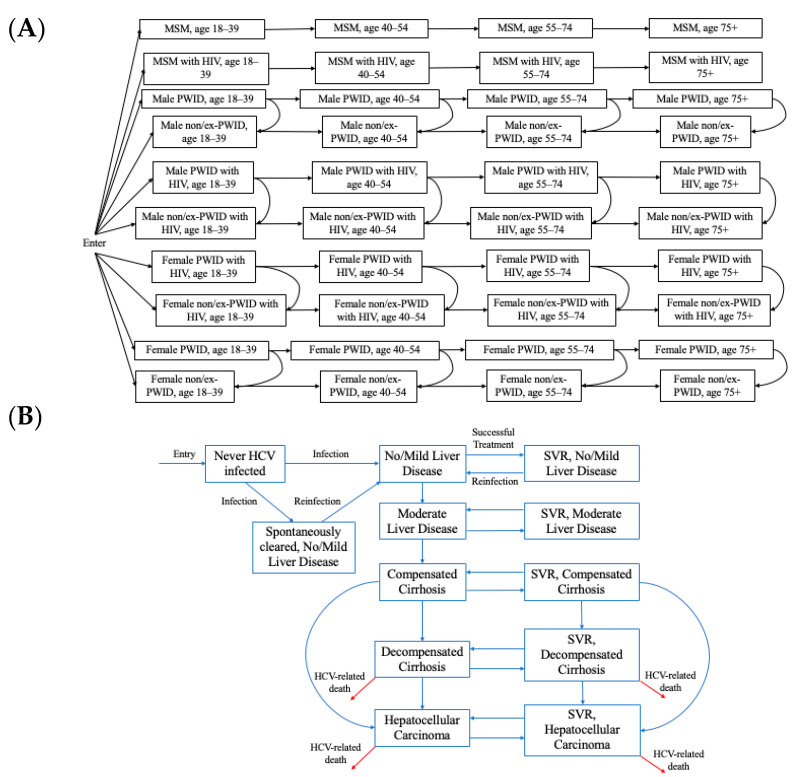
Hepatitis C virus (HCV) transmission and disease progression model schematics for the (**A**) population groups and (**B**) HCV infection and disease progression. SVR: sustained viral response. MSM: men who have sex with men. PWID: people who inject drugs.

We adapted a previously developed dynamic, deterministic compartmental model of HCV transmission and disease progression among adults in the COSD [7]. The model simulates the entire adult population of the COSD, stratified by transmission risk (the model previously included people who inject drugs (PWID) and men who have sex with men (MSM) as it was focused only on transmission and was subsequently extended to incorporate former or never PWID (ex/non PWID) to track disease burden in the entire population). The model is open, with individuals entering at age 18 into one of the population groups (MSM, PWID, ex/non-PWID) and leaving the model due to age-specific mortality. Additionally, PWID can permanently cease injecting and move into the ex/non-PWID compartments. Each group is also stratified by age (18–39, 40–54, 55–74, and 75+), gender (male/female), HIV status (susceptible, infected) (Figure 1A), and HCV disease stage (Figure 1B). The hepatitis C infection and disease stages are: (i) susceptible, (ii) spontaneous clearance from no/mild liver disease, (iii) sustained viral response (SVR) from no/mild liver disease (iv) susceptible moderate liver disease, (v) susceptible compensated cirrhosis, (vi) susceptible decompensated cirrhosis, (vii) susceptible hepatocellular carcinoma, (viii) no/mild liver disease, (ix) moderate liver disease, (x) compensated cirrhosis, (xi) decompensated cirrhosis, and (xii) hepatocellular carcinoma. We assume HIV status is a fixed characteristic; the model does not simulate HIV transmission dynamically due to the stable HIV prevalence among PWID and MSM in the COSD [8,9].

The model is dynamic, such that susceptible individuals may become infected (or reinfected) with HCV at a rate proportional to the prevalence of active HCV infection among their risk group, which can change over time as people are treated and cured. In the model, HCV transmission is simulated among PWID and MSM separately based on phylogenetic analyses in other settings indicating that these epidemics are distinct [10]. As such, although MSM may inject drugs, we classify these individuals as MSM and assume they inject with other MSM. The model also assumes assortative mixing among MSM by HIV status. People with HIV are assumed to have elevated susceptibility and transmissibility for HCV compared to those without HIV [11,12]. We assumed a time-varying elevated risk of transmission among young PWID (aged 18–39) compared to older PWID, which was necessary to recreate observed HCV prevalence trends by age among PWID in San Diego [13] and also supported by data on higher overdose rates among this group in the past decade [14].

Once acutely infected, individuals can either transition to the no/mild liver disease compartment or spontaneously clear HCV infection and move to the spontaneous clearance from no/mild liver disease compartment. Spontaneous clearance is reduced for those with HIV. Individuals with chronic infection continue to progress through the disease stages unless successfully treated. Treatment occurs at a rate that varies by population and time. Those who have been successfully treated move into the equivalent SVR stage and are susceptible to reinfection at a risk similar to primary infection. Successful treatment stops any HCV-related disease progression unless an individual has already reached the compensated cirrhosis stage or beyond, whereupon disease progression occurs at a slower rate compared to those without SVR [15,16]. HCV-related mortality occurs from the decompensated cirrhosis compartments and hepatocellular carcinoma compartments, and we include a scaling factor to reduce liver-related mortality to account for liver transplant (which is obtained through model calibration). All individuals are at risk of background (non-HCV-related mortality), and PWID experience an elevated risk of mortality due to drug-related causes.

### 2.2. Model Parameterization and Calibration

The model was parameterized from the published literature for aspects such as HCV natural history, treatment SVR, and age-related mortality (see Supplementary Table S1). The model was calibrated to detailed epidemiological data from several local sources (Table 1), with detailed PWID data obtained from a longitudinal cohort of PWID in San Diego (La Frontera Cohort, PI Strathdee) [13]. HCV treatment among PWID was estimated to be negligible from La Frontera [13], where among PWID residing in San Diego, HCV RNA prevalence among Ab+ PWID was 76%, similar to that expected with spontaneous clearance only and no treatment. This was consistent with self-reported exposure to HCV treatment (unpublished, La Frontera). As we did not have data on HCV treatment among HCV-infected MSM without HIV, we assumed no treatment based on little historical surveillance in this group. For the remaining groups (ex/non PWID without HIV), HCV treatment rates were estimated from general population care cascade studies in San Francisco and nationally (approximately 5%/year) [17,18]. Time-varying HCV treatment rates among people coinfected with HCV and HIV were obtained through model calibration (described in our previous publication [7] and below). We simulated a piece-wise time-varying treatment function to represent different treatment eras: 1996–2010 (pegylated interferon (IFN) plus ribavirin era), 2011–2017 (first generation of direct-acting antiviral (DAA) therapies), and 2018–2021 (broadly available IFN-free DAAs).

We used a two-step calibration process for the model. First, we used a simplified submodel (simulating PWID and MSM only, neglecting ex/non-PWID) to determine transmission and treatment-related parameters for these groups. This allowed us to seed the epidemic in 1955 and run the model to achieve equilibrium prior to the recent scale-up of treatment and changes in transmission risk. Then, we inputted these calibrated transmission and treatment parameters into the full model (simulating all risk groups), which was initialized in 2015 based on our recent burden estimation (for non-PWID) and outputs from the submodel (for PWID and MSM). Using this full model, we then ran a secondary calibration to determine HCV-related mortality parameters based on recent mortality trends. The final calibrated model thus was consistent with epidemiological data on both HCV prevalence and mortality. Details of the full model and the submodel used for calibration are found in the Supplementary Materials. Therefore, the final full model was calibrated to data on HCV seroprevalence in 2015 among MSM (4.6% among all MSM, 16.5% among MSM with HIV) [5,22]; HCV viremia prevalence among HCV seropositive people coinfected with HIV of 42.1% (2010), 18.5% (2018), and 8.5% (2021) [19]; the number of PWID in 2007 [5,20]; HCV seroprevalence of 46% and 36% among young PWID (aged 18–39) and older PWID (aged 40–74) in 2021, respectively [13]; primary HCV incidence rate among PWID of 17.14 per 100 person-years in 2021 [13]; and total HCV-related deaths in 2015 (290) and 2019 (320) [21]. Calibration was obtained by varying the following parameters: transmission rate among MSM, transmission rate among PWID, degree of assortative mixing among MSM by HIV status, annual treatment rates among HIV/HCV co-infected individuals (1996–2010, 2011–2017, and 2018–2021), the relative risk of transmission among young PWID (aged 18–39), start year of increased risk among young PWID, HCV-related death rate scaling factor, and the proportion of HCV Ab+ non-PWID with previous successful treatment in 2015.

For each calibration, to capture the uncertainty in input parameters, a sample of 100 parameter sets was drawn from uncertainty distributions for each unknown parameter. We assigned wide prior bounds to the unknown parameters and assessed our posterior estimates to ensure our priors were sufficiently wide. These sampled parameter sets were then used to generate 100 model fits to the observed data. Calibration was achieved using a least-squares minimization solver in MATLAB version R2023a (*lsqnonlin* function) with multiple start points (MultiStart) to ensure a global minimum was found. From the full calibrated model fits, runs were then excluded if the fits fell outside the 95% C.I. of the calibration data for HCV seroprevalence in 2021 among (i) young PWID (aged 18–39) and (ii) older PWID (aged 40–54) [13], generating a total of 76 model fits to the data.

### 2.3. Modeled Scenarios

The calibrated model fits were used to simulate HCV incidence and HCV-related mortality from 2015 to 2030. We simulated a status quo scenario and also scenarios of combination scale-up of treatment and reductions in injecting transmission risk based on recent authorization of county founding for syringe service programs (SSP), which we believe will likely reduce transmission risk.

- Scenario 1 (status quo): Continuation of current treatment rates (65.1% among people coinfected with HCV and HIV, 0% among PWID and MSM without HIV, and 5%/year among ex/non-PWID without HIV).
- Scenario 2 (scale-up to meet the 80% incidence elimination goal without injecting transmission risk reduction from 2024 onward): Scale-up of HCV treatment among people without HIV from 2024 onward to a rate that achieves an 80% reduction in new HCV infections from 2030 compared to 2015. This rate was determined through model calibration. Treatment among people coinfected with HCV and HIV is held constant at 65%/year.
- Scenario 3 (scale-up to meet the 80% incidence elimination goal with 25% injecting transmission risk reduction from 2024 onward): Reduction in injecting transmission risk by 25% from 2024 onward combined with scale-up of HCV treatment among people without HIV from 2024 onward to a rate that achieves an 80% reduction in new HCV infections from 2030 compared to 2015. This rate was determined through model calibration. Treatment among people coinfected with HCV and HIV is held constant at 65%/year.
- Scenario 4 (scale-up to meet the 80% incidence elimination goal with 50% injecting transmission risk reduction from 2024 onward): Reduction in injecting transmission risk by 50% from 2024 onward combined with scale-up of HCV treatment among people without HIV from 2024 onward to a rate that achieves an 80% reduction in new HCV infections from 2030 compared to 2015. This rate was determined through model calibration. Treatment among people coinfected with HCV and HIV is held constant at 65%/year.

### 2.4. Sensitivity and Uncertainty Analyses

We performed a partial rank coefficient correlation (PRCC) uncertainty analysis to understand how sensitive the model prediction of HCV treatment required to achieve elimination without any transmission risk reduction is to uncertainty in underlying parameters.

## 3. Results

**Figure 2:**
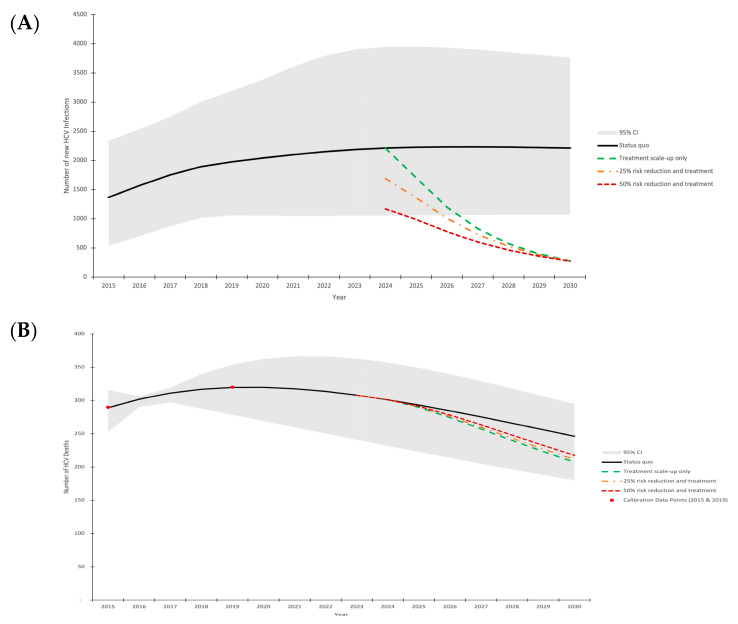
Model projections of annual HCV incidence (**A**) and HCV-related mortality (**B**) in the COSD (2015–2030). Mean model projections (lines), with shading denoting the 95% uncertainty interval around the status quo scenario. Scenarios shown are: (1) status quo treatment (black solid line); (2) treatment scale-up to achieve 80% incidence reduction without injecting transmission risk reduction from 2024 onward (green dashed line); (3) treatment scale-up to achieve 80% incidence reduction with 25% injecting transmission risk reduction among PWID from 2024 onward (orange dash dot line); (4) treatment scale-up to achieve 80% incidence reduction with 50% injecting transmission risk reduction among all PWID from 2024 onward (dark red square dot line).

The calibrated model runs (n = 76) fit well to the data (Table 1). The model projects 1366 [95% C.I.: 536–2340] incident HCV infections in 2015 in the COSD, with a steady increase to 2211 [95% C.I.: 1054–3949] new HCV infections in 2024 (Figure 2A). Similarly, the model projects 289 [95% C.I.: 253, 316] HCV-related deaths in 2015, with a slight increase to 301 [95% C.I.: 232–357] HCV-related deaths in 2024 (Figure 2B).

Without any intervention scale-up, our model projects an increase in annual new HCV infections to 2213 [95% C.I.: 1069–3763] in 2030, failing to meet the elimination incidence target and resulting in an increase in annual incidence by a relative 91% [95% C.I.: 1–355%] between 2015 and 2030, alongside increasing incidence rates (Supplementary Figure S1). Although HCV-related deaths are expected to decrease to 246 [95% C.I.: 180–295] in 2030, this would fail to meet the 65% reduction mortality target, resulting in a relative decrease of 14% [95% C.I.: −18–45%] between 2015 and 2030.

### 3.1. Treatment Needed to Reach Elimination

In order to reach the 80% incidence reduction elimination target, the model indicates that without any harm reduction scale-up or injecting transmission risk reduction, treatment would need to be scaled-up to 60%/year [95% C.I.: 39–98%] among people with HCV who do not have HIV (among people coinfected with HCV and HIV, the baseline rate is already 65%/YEAR). This level of treatment achieves the incidence target (declining to 273 [95% C.I.: 107–468] new infections in 2030) but only results in a moderate decline in HCV-related deaths (declining to 207 [95% C.I.: 153–258] in 2030, a 28% reduction compared to 2015). Greater treatment rates, particularly among those most at risk of HCV-related mortality, would be required to achieve larger reductions in mortality.

**Figure 3:**
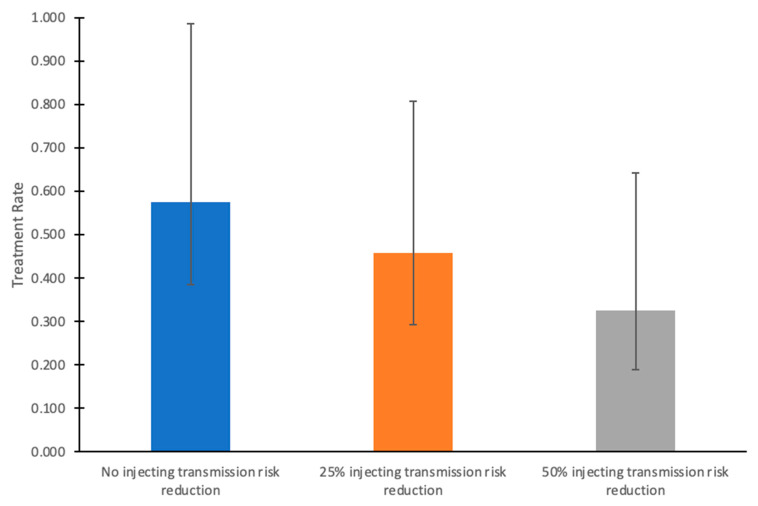
Annual HCV treatment rates required to achieve the HCV incidence elimination goal by 2030 among HCV-infected people without HIV, given varying levels of injecting transmission risk reduction from 2024 onward in the COSD. In these scenarios, HCV treatment rates among people coinfected with HCV and HIV remain at their baseline (65%/year). Bars indicate mean values, and whiskers denote the lower (2.5th percentile) and upper (97.5th percentile) bounds from the simulations.

If injecting-related transmission risk reductions were achieved alongside HCV treatment scale-up from 2024 onward (such as through scale-up of harm reduction interventions), then lower treatment rates are required to reach the HCV incidence target (Figure 3). With 25% and 50% injecting transmission risk reductions among PWID, then only 48%/year [95% C.I.: 29–80%] and 35%/year [95% C.I.: 19–63%] treatment rates among those without HIV are required, respectively, to reach the incidence targets.

### 3.2. Uncertainty Analysis

The predicted treatment rate required to achieve elimination is most sensitive to the PWID transmission rate and the relative risk of transmission among young PWID compared to old PWID, which accounted for 44% and 18% of the variance in the outcome, respectively (Supplementary Figure S2). The remainder of the parameters contributed <10% to uncertainty.

## 4. Discussion

Our modeling indicates that the COSD is likely not on track to reach the *Eliminate Hepatitis C Initiative’s* incidence or mortality targets, but achieving the incidence target is possible if treatment rates are scaled-up among HCV-infected individuals to rates that have been achieved among people coinfected with HIV and HCV in the COSD. As such, we believe elimination is achievable but requires committed funding (as was achieved through the Ryan White program for people with HIV) and comprehensive testing, linkage, and treatment programs alongside harm reduction initiatives (i.e., syringe service programs; SSPs). The recent FDA approval for point-of-care HCV RNA tests in the United States is an important opportunity to expand and innovate initiatives to diagnose individuals and link them with care [23]. However, these tests are only available to adults aged 22 and older, which excludes a significant proportion of PWID. Additionally, these initiatives urgently require committed funding. Congressional approval of funding for the White House HCV elimination initiative is urgently needed to provide funding for novel point-of-care diagnostics and treatment. Even with this influx of funding, it is essential that testing and treatment initiatives are focused on identifying those with ongoing transmission risk, such as PWID. Providing HCV testing and treatment in community settings (like syringe service programs (SSPs) and overdose prevention centers) is an effective way to reach PWID and their partners and provide them with low-barrier care.

Further, we find that a combination approach of scaling up both treatment and harm-reduction initiatives for PWID could reduce the number needed to treat to achieve the incidence elimination goals. Evidence-based harm reduction interventions, such as opiate agonist therapy (OAT) and SSPs, have been shown to reduce the risk of HCV transmission by up to 80% if used in combination [24]. Although the uptake of OAT among PWID is low in the COSD (<15% recently on OAT in the La Frontera cohort, personal communication), initiatives during the onset of the COVID-19 pandemic (such as allowing take-home doses and eliminating X-waivers required for prescribing buprenorphine) may serve to increase uptake in the future. Since 2020, the number of state-authorized SSPs has quadrupled in the COSD, which may increase syringe coverage and reduce syringe sharing among PWID in the region. However, longitudinal monitoring is necessary to understand the impact of this additional scale-up of SSP services on HCV incidence and subsequent mortality.

Our work is consistent with other analyses indicating that the U.S. is not on track to reach its incidence elimination targets, but that the incidence elimination goal can be achieved with a combined harm reduction and treatment approach [25,26]. However, our work has a number of key uncertainties. First, there is uncertainty surrounding changing drug markets and associated drug practices in the COSD, as well as subsequent HCV risk. For example, recent shifts from injecting to smoking among PWID in San Francisco and San Diego have been observed, alongside an increasing penetration of fentanyl in the drug supply. However, the impact of these changes on HCV incidence is unclear. On the one hand, less injecting could lead to less HCV transmission, but our recent analysis indicates that fentanyl use predicts HCV seroconversion, even after adjusting for syringe sharing [27]. Future epidemiological and modeling work using longitudinal data from the cohort of PWID in San Diego will enable more detailed characterization of these trends over time and the resulting impact on HCV and mortality. Second, there is uncertainty in HCV treatment rates among MSM without HIV and former PWID in the COSD. The COSD is currently in the process of developing a comprehensive HCV treatment database, which will enable the monitoring of HCV treatment and provide local data to inform updated modeling. Third, it is important that our model is prospectively validated against future surveillance data and updated accordingly. These limitations notwithstanding, we believe that our analysis provides an important data-based foundation for programmatic targets to ensure HCV elimination is achieved in the COSD.

Modeling studies like ours are useful in estimating HCV incidence (a key WHO elimination target) in the absence of direct incidence estimation through retesting of people at risk, which can be highly resource-intensive [28]. In settings where HCV incidence cannot be monitored, surveillance of chronic HCV prevalence or monitoring trends in HCV prevention and treatment combined with modeling (as presented in this study) can be a reliable alternative [28].

In conclusion, the COSD is not on track to reach its HCV elimination targets but could achieve them by 2030 with committed funding, scaled-up HCV testing, treatment, harm reduction initiatives, and building on local micro-elimination efforts among people coinfected with HCV and HIV [29,30]. Indeed, as 2030 approaches, despite progress in select countries, the majority of countries are not on track to reach the WHO incidence and mortality targets [31]. In the many settings where the majority of transmission is among PWID, more concerted efforts are needed to provide enhanced prevention, testing, and treatment among PWID to substantially reduce HCV incidence [32].

## Figures and Tables

**Table 1:** HCV calibration data for the county of San Diego. PWID: people who inject drugs. MSM: men who have sex with men.

	Year	Observed Data Used for Model Calibration [95% C.I.]	Calibrated Model Output [95% C.I.]	Reference
HCV seroprevalence among MSM coinfected with HIV	2015	0.165 [0.155, 0.176]	0.157 [0.122, 0.172]	[5]
HCV seroprevalence among all MSM	2015	0.046 [0.030, 0.061]	0.071 [0.050, 0.103]	[5]
HCV seroprevalence among PWID [age 18–39]	2021	0.46 [0.39, 0.53]	0.450 [0.407, 0.479]	La Frontera PWID cohort with residence in SD, unpublished [13]
HCV seroprevalence among PWID [age 40–74]	2021	0.36 [0.30, 0.43]	0.395 [0.363, 0.428]	La Frontera PWID cohort with residence in SD, unpublished [13]
HCV viremia prevalence among HCV seropositive people coinfected with HIV	2010	0.3091 [0.2705, 0.3506]	0.3082 [0.2633, 0.3400]	[19]
	2018	0.1849 [0.1534, 0.2212]	0.193 [0.159, 0.266]	[19]
	2021	0.0857 [0.0636, 0.1146]	0.095 [0.082, 0.118]	[19]
Number of PWID	2007	24,991	30,075 [25,055, 37,948]	[5,20]
HCV primary incidence rate among PWID (per 100 person-years)	2021	17.14 [12.52, 21.75]	9.592 [5.184, 16.561]	La Frontera PWID cohort with residence in SD, unpublished [13]
HCV-related deaths	2015	290	289 [253, 316]	CDC [21]

## Data Availability

Available on request from the authors.

## Supplementary Materials

The following supporting information can be downloaded at: https://www.mdpi.com/article/10.3390/v16121819/s1, Figure S1: Model projections of HCV incidence rate (per 100 person-years) among PWID in San Diego County (2015–2030); Figure S2: PRCC analysis on the HCV treatment rate required for elimination without transmission risk reductions; Table S1: Model Parameters, Sampling Distributions, and Sources. References [33,34,35,36,37,38,39,40,41,42,43,44,45,46,47,48] are cited in the Supplementary Materials.
