# Supplementary material for: Is San Diego California on Track to Reach HCV Elimination? A Modeling Analysis of Combination Prevention Strategies
Source: Viruses. 2024 Nov 22;16(12):1819. doi: 10.3390/v16121819 (PMC11680419; doi:10.3390/v16121819)
Supplement: Supplementary file 1 [file viruses-16-01819-s001.zip › viruses-3243016-supplementary.pdf]

SUPPLEMENTARY INFORMATION

TABLE OF CONTENTS

A. Full model equations……………………………………………………………………2
B. Full model parameterization data ………………………..…………………………8
C. Model calibration process and description of submodel used to estimate a subset of parameters……..……………………………………………………..……………13
D. Submodel equations……..…………..…………………………………..……………17
E. Supplementary figures……..……………..……………………..………..……..………20

FULL MODEL EQUATIONS

A description of all values is provided in the tables above and the model schematics illustrate the model flows (Figures 1a & 1b).

For the below equations:

i = age stage (1-4), where,
i = 1: 18-39-year olds
i = 2: 40-54-year olds
i = 3: 55-74-year olds
i = 4: 75+ year olds

j = population sub-type (1-10), where,
j = 1: MSM
j = 2: MSM HIV+
j = 3: PWID Male
j = 4: Non/Ex-PWID Male
j = 5: PWID HIV+ Male
j = 6: Non/Ex-PWID HIV+ Male
j = 7: PWID Female
j = 8: Non/Ex-PWID Female
j = 9: PWID HIV+ Female
j = 10: Non/Ex-PWID HIV+ Female

X_n denotes the infection/disease stages, where,
n = infection/disease stages, and
n = 1: Susceptible (X_1)
n = 2: Spontaneous Clearance from no/mild liver disease (X_2)
n = 3: SVR from no/mild liver disease (X_3)
n = 4: Susceptible Moderate Liver Disease (X_4)
n = 5: Susceptible Compensated Cirrhosis (X_5)
n = 6: Susceptible Decompensated Cirrhosis (X_6)
n = 7: Susceptible Hepatocellular Carcinoma (X_7)
n = 8: No/Mild Liver Disease (X_8)
n = 9: Moderate Liver Disease (X_9)
n = 10: Compensated Cirrhosis (X_{10})
n = 11: Decompensated Cirrhosis (X_{11})
n = 12: Hepatocellular Carcinoma (X_{12})

φ_i = population ageing rate, where,
φ_1 = ageing rate from i = 1 to i = 2
φ_2 = ageing rate from i = 2 to i = 3
φ_3 = ageing rate from i = 3 to i = 4

$\varphi_0 = \varphi_4 = 0$

μ_i = Background mortality rate, where the rate is dependent on age

δ^j = Overdose mortality rate, where $\delta^{j=1,2,4,6,8,10} = 0$

$$\delta^{j=3,5,7,9} = \delta \; if \; t \leq 2017$$
$$\delta^{j=3,5,7,9} = \delta * \varepsilon \; if \; t = 2018$$
$$\delta^{j=3,5,7,9} = \delta * 2\varepsilon \; if \; t = 2019$$
$$\delta^{j=3,5,7,9} = \delta * 3\varepsilon \; if \; t = 2020$$
$$\delta^{j=3,5,7,9} = \delta * 4\varepsilon \; if \; t \geq 2021$$

ν_n = HCV-related death rate, where, $\nu_{n=1,2,3,4,5,8,9,10} = 0$

ω = Reduction in HCV related mortality among chronically infected HCV individuals due to liver transplant

$trt_{i,x}^{j}(t)$ = treatment proportion, where,
$trt_{i,x=1}^{j}(t)$ = proportion of individuals treated from no/mild liver disease ($X_{i,n=8}^{j}$)
$trt_{i,x=2}^{j}(t)$ = proportion of individuals treated from moderate liver disease ($X_{i,n=9}^{j}$)
$trt_{i,x=3}^{j}(t)$ = proportion of individuals treated from compensated cirrhosis ($X_{i,n=10}^{j}$)
$trt_{i,x=4}^{j}(t)$ = proportion of individuals treated from decompensated cirrhosis ($X_{i,n=11}^{j}$)
$trt_{i,x=5}^{j}(t)$ = proportion of individuals treated from HCC ($X_{i,n=12}^{j}$)

For 2017-2021 we simulated a weighted treatment rate between UCSD and non-UCSD clinics (with UCSD clinics providing care for an estimated κ=26% of people with HIV in San Diego), using historical treatment data from UCSD clinics and calibrating the non-UCSD rate to achieve the observed chronic prevalence declines among people with HIV, such that

$$trt_{i,x}^{j=2,5,6,9,10} = tUCSD * \kappa + tNON * (1 - \kappa) \quad if \;\; 2017 \;\leq t \; < 2021$$

HR_{DC} = Hazard ratio of progression to decompensated cirrhosis after SVR compared to no SVR
HR_{HCC} = Hazard ratio of progression to HCC after SVR compared to no SVR

rop_x = rate of disease progression, where,
$rop_{x=1}$ = rate of disease progression from ($X_{i,n=8}^{j}$) to ($X_{i,n=9}^{j}$)
$rop_{x=2}$ = rate of disease progression from ($X_{i,n=9}^{j}$) to ($X_{i,n=10}^{j}$)
$rop_{x=3}$ = rate of disease progression from ($X_{i,n=10}^{j}$) to ($X_{i,n=11}^{j}$)
$rop_{x=4}$ = rate of disease progression from ($X_{i,n=10}^{j}$) to ($X_{i,n=12}^{j}$)

$rop_{x=5}$ = rate of disease progression from $(X^j_{i,n=11})$ to $(X^j_{i,n=12})$
$rop_{x=6}$ = rate of disease progression from $(X^j_{i,n=5})$ to $(X^j_{i,n=6})$ = $HR_{DC} * rop_{x=3}$
$rop_{x=7}$ = rate of disease progression from $(X^j_{i,n=5})$ to $(X^j_{i,n=7})$ = $HR_{DC} * rop_{x=4}$
$rop_{x=8}$ = rate of disease progression from $(X^j_{i,n=6})$ to $(X^j_{i,n=7})$ = $HR_{HCC} * rop_{x=5}$

p^j = proportion of individuals that spontaneously clear HCV infection which varies by HIV status:

$$\begin{cases} p^{j=1,3,4,7,8} = p \\ p^{j=2,5,6,9,10} = \text{pHIV} \end{cases}$$

$c^j\ (t)$ = proportion of individuals that attain sustained viral response (SVR) which varies by time and HIV status:

$$\begin{cases} c^{j=1,3,4,7,8} = c(t) \\ c^{j=2,5,6,9,10} = \text{cHIV(t)} \end{cases}$$

RR_{HIV_tr} = relative risk of transmission among HIV+ individuals
RR_{HIV_s} = relative risk of susceptibility among HIV+ individuals

As detailed in the main text, we simulate a time-varying risk of transmission among PWID in the youngest age category (age 18-39) compared to the older categories, with the year that this elevated risk starts found through model calibration:

$$\begin{cases} RR_{yPWID} = 1\ if\ t < YearRR \\ RR_{yPWID} = RR_{young}\ if\ t \geq YearRR \end{cases}$$

m = degree of assortative mixing among HIV+ and non-HIV+ MSM

ψ^j = rate of transition from PWID to ex-PWID, where,

$$\begin{cases} \psi_i^{j=0,1,2,4,6,8,10} = 0 \\ \psi_i^{j=3,5,7,9} = \psi \end{cases}$$

The entry rate is denoted by the term, α. We replaced all deaths with entries, excluding HCV and excess drug-related deaths from 2017 onwards (where the rate increases), such that the population is stable without HCV and without excess drug-related mortality from 2017 onwards.

Force of Infection Terms
People in HIV+ compartments having an additional relative risk term of transmissibility (RR_{HIV_tr}), and susceptibility (RR_{HIV_s}).

For MSM (*j* = 1, 2 and *i* = 1, 2, 3, 4)

$$\frac{I\_MSM\_HIV(t)}{N\_MSM\_HIV(t)} = \frac{\sum_{i=1}^{4} \sum_{n=8}^{12}(RR_{HIV\_tr} * X_{i,n}^{j=2})}{\sum_{i=1}^{4} \sum_{n=8}^{12}(RR_{HIV\_tr} * X_{i,n}^{j=2}) + \sum_{i=1}^{4} \sum_{n=1}^{7}(RR_{HIV_{tr}} * X_{i,n}^{j=2})}$$

$$\frac{I\_MSM\_HIVneg(t)}{N\_MSM\_HIVneg(t)} = \frac{\sum_{i=1}^{4} \sum_{n=8}^{12}(X_{i,n}^{j=1})}{\sum_{i=1}^{4} \sum_{n=8}^{12}(X_{i,n}^{j=1}) + \sum_{i=1}^{4} \sum_{n=1}^{7}(X_{i,n}^{j=1})}$$

$$\frac{I\_MSM(t)}{N\_MSM(t)} = \frac{\sum_{i=1}^{4} \sum_{n=8}^{12}(X_{i,n}^{j=1} + RR_{HIV\_tr} * X_{i,n}^{j=2})}{\sum_{i=1}^{4} \sum_{n=8}^{12}(X_{i,n}^{j=1} + RR_{HIV\_tr} * X_{i,n}^{j=2}) + \sum_{i=1}^{4} \sum_{n=1}^{7}(X_{i,n}^{j=1} + RR_{HIV_{tr}} * X_{i,n}^{j=2})}$$

Force of infection for MSM HIV-: $foi_i^{j=1} = \beta_{MSM} * \left(m * \left[\frac{I_{MSM_{HIVneg(t)}}}{N_{MSM_{HIVneg(t)}}}\right] + (1-m) * \left[\frac{I_{MSM(t)}}{N_{MSM(t)}}\right]\right)$

Force of infection for MSM HIV+: $foi_i^{j=2} = \beta_{MSM} * RR_{HIV_s} * \left(m * \left[\frac{I_{MSM_{HIV(t)}}}{N_{MSM_{HIV(t)}}}\right] + (1-m) * \left[\frac{I_{MSM(t)}}{N_{MSM(t)}}\right]\right)$

For PWID (*j* =3, 5, 7, 9 and *i* = 1, 2, 3, 4)

$$\frac{I\_PWID(t)}{N\_PWID(t)} = \frac{\sum_{n=8}^{12}(X_{i,n}^{j=3} + X_{i,n}^{j=7} + RR_{HIV\_tr} * (X_{i,n}^{j=5} + X_{i,n}^{j=9}))}{\sum_{n=8}^{12}(X_{i,n}^{j=3} + X_{i,n}^{j=7} + RR_{HIV\_tr} * (X_{i,n}^{j=5} + X_{i,n}^{j=9})) + \sum_{n=1}^{7}(X_{i,n}^{j=3} + X_{i,n}^{j=7} + RR_{HIV\_tr} * (X_{i,n}^{j=5} + X_{i,n}^{j=9})}$$

Force of infection for young PWID Male HIV-: $foi_{i=1}^{j=3} = \beta_{PWID\_m} * RR_{yPWID}(t) * \left[\frac{I\_PWID(t)}{N\_PWID(t)}\right]$

Force of infection for young PWID Male HIV+: $foi_{i=1}^{j=5} = \beta_{PWID\_m} * RR_{yPWID}(t) * RR_{HIV_s} * \left[\frac{I\_PWID(t)}{N\_PWID(t)}\right]$

Force of infection for young PWID Female HIV-: $foi_{i=1}^{j=7} = \beta_{PWID\_f} * RR_{yPWID}(t) * \left[\frac{I\_PWID(t)}{N\_PWID(t)}\right]$

Force of infection for young PWID Female HIV+: $foi_{i=1}^{j=9} = \beta_{PWID\_f} * RR_{yPWID}(t) * RR_{HIV_s} * \left[\frac{I\_PWID(t)}{N\_PWID(t)}\right]$

Force of infection for older PWID Male HIV-: $foi_{i=2,3,4}^{j=3} = \beta_{PWID\_m} * \left[\frac{I\_PWID(t)}{N\_PWID(t)}\right]$

Force of infection for older PWID Male HIV+: $foi_{i=2,3,4}^{j=5} = \beta_{PWID\_m} * RR_{HIV_s} * \left[\frac{I\_PWID(t)}{N\_PWID(t)}\right]$

Force of infection for older PWID Female HIV-: $foi_{i=2,3,4}^{j=7} = \beta_{PWID\_f} * \left[\frac{I\_PWID(t)}{N\_PWID(t)}\right]$

Force of infection for older PWID Female HIV+: $foi_{i=2,3,4}^{j=9} = \beta_{PWID\_f} * RR_{HIV_s} * \left[\frac{I\_PWID(t)}{N\_PWID(t)}\right]$

For Non/Ex-PWID (*j* = 4, 6, 8, 10 and *i* = 1, 2, 3, 4)
Force of infection for Non/Ex-PWID: $foi_i^{j=4,6,8,10} = 0$

Model Equations
The model equations are as follows

$$\frac{dX_{n=1}}{dt} = \alpha_{i=1}^{j} - X_{i,n=1}^{j} * \left(foi_i^j * p^j + foi_i^j * \left(1 - p^j\right) + \mu_i + \varphi_i + \psi_i^j + \delta^j \right) + X_{i,n=1}^{j-1} * \psi_i^{j-1} + X_{i-1,n=1}^{j} * \varphi_{i-1}$$

$$\frac{dX_{n=2}}{dt} = X_{i,n=1}^{j} * foi_i^j * p^j - X_{i,n=2}^{j} * \left(foi_i^j * \left(1 - p^j\right) + \mu_i + \varphi_i + \psi_i^j + \delta^j \right) + X_{i,n=1}^{j-1} * \psi_i^{j-1} + X_{i-1,n=2}^{j} * \varphi_{i-1}$$

$$\frac{dX_{n=3}}{dt} = -X_{i,n=3}^{j} * \left(foi_i^j * \left(1 - p^j\right) + \mu_i + \varphi_i + \psi_i^j + \delta^j \right) + X_{i,n=8}^{j} * trt_{i,x=1}^{j}(t) * c^j (t) + X_{i,n=1}^{j-1} * \psi_i^{j-1} + X_{i-1,n=3}^{j} * \varphi_{i-1}$$

$$\frac{dX_{n=4}}{dt} = -X_{i,n=4}^{j} * \left(foi_i^j * \left(1 - p^j\right) + \mu_i + \varphi_i + \psi_i^j + \delta^j \right) + X_{i,n=9}^{j} * trt_{i,x=2}^{j}(t) * c^j (t) + X_{i,n=1}^{j-1} * \psi_i^{j-1} + X_{i-1,n=4}^{j} * \varphi_{i-1}$$

$$\frac{dX_{n=5}}{dt} = -X_{i,n=5}^{j} * \left(foi_i^j * \left(1 - p^j\right) + rop_{x=6} + rop_{x=7} + \mu_i + \varphi_i + \psi_i^j + \delta^j \right) + X_{i,n=10}^{j} * trt_{i,x=3}^{j}(t) * c^j (t) + X_{i,n=1}^{j-1} * \psi_i^{j-1} + X_{i-1,n=5}^{j} * \varphi_{i-1}$$

$$\frac{dX_{n=6}}{dt} = -X_{i,n=6}^{j} * \left(foi_i^j * \left(1 - p^j\right) + rop_{x=8} + (\nu_n * \omega) + \mu_i + \varphi_i + \psi_i^j + \delta^j \right) + X_{i,n=5}^{j} * rop_{x=6} + X_{i,n=11}^{j} * trt_{i,x=4}^{j}(t) * c^j (t) + X_{i,n=1}^{j-1} * \psi_i^{j-1} + X_{i-1,n=6}^{j} * \varphi_{i-1}$$

$$\frac{dX_{n=7}}{dt} = -X_{i,n=7}^{j} * \left(foi_i^j * \left(1 - p^j\right) + (\nu_n * \omega) + \mu_i + \varphi_i + \psi_i^j + \delta^j \right) + X_{i,n=12}^{j} * trt_{i,x=5}^{j}(t) * c^j (t) + X_{i,n=5}^{j} * rop_{x=7} + X_{i,n=6}^{j} * rop_{x=8} + X_{i,n=1}^{j-1} * \psi_i^{j-1} + X_{i-1,n=7}^{j} * \varphi_{i-1}$$

$$\frac{dX_{n=8}}{dt} = foi_i^j * \left(1 - p^j\right) * \left(X_{i,n=1}^{j} + X_{i,n=2}^{j} + X_{i,n=3}^{j}\right) - X_{i,n=8}^{j} * \left(trt_{i,x=1}^{j}(t) * c^j (t) + rop_{x=1} + \mu_i + \varphi_i + \psi_i^j + \delta^j \right) + X_{i,n=1}^{j-1} * \psi_i^{j-1} + X_{i-1,n=8}^{j} * \varphi_{i-1}$$

$$\frac{dX_{n=9}}{dt} = X_{i,n=4}^{j} * foi_i^j * \left(1 - p^j\right) - X_{i,n=9}^{j} * \left(trt_{i,x=2}^{j}(t) * c^j (t) + rop_{x=2} + \mu_i + \varphi_i + \psi_i^j + \delta^j \right) + X_{i,n=8}^{j} * rop_{x=1} + X_{i,n=1}^{j-1} * \psi_i^{j-1} + X_{i-1,n=9}^{j} * \varphi_{i-1}$$

$$\frac{dX_{n=10}}{dt} = X_{i,n=5}^{j} * foi_i^j * \left(1 - p^j\right) - X_{i,n=10}^{j} * \left(trt_{i,x=3}^{j}(t) * c^j (t) + rop_{x=3} + rop_{x=4} + \mu_i + \varphi_i + \psi_i^j + \delta^j \right) + X_{i,n=9}^{j} * rop_{x=2} + X_{i,n=1}^{j-1} * \psi_i^{j-1} + X_{i-1,n=10}^{j} * \varphi_{i-1}$$

$$\frac{dX_{n=11}}{dt} = X_{i,n=6}^{j} * foi_{i}^{j} * \left(1 - p^{j}\right) - X_{i,n=11}^{j} * \left(trt_{i,x=4}^{j}(t) * c^{j}\ (t) + rop_{x=5} + (\nu_{n}\ * \omega\ ) + \mu_{i}\ + \varphi_{i} + \psi_{i}^{j} + \delta^{j}\ \right) + X_{i,n=10}^{j} * rop_{x=3} + X_{i,n=1}^{j-1} * \psi_{i}^{j-1} + X_{i-1,n=11}^{j} * \varphi_{i-1}$$

$$\frac{dX_{n=12}}{dt} = X_{i,n=7}^{j} * foi_{i}^{j} * \left(1 - p^{j}\right) - X_{i,n=12}^{j} * \left(trt_{i,x=5}^{j}(t) * c^{j}\ (t) +\ (\nu_{n}\ * \omega\ ) + \mu_{i}\ + \varphi_{i} + \psi_{i}^{j} + \delta^{j}\ \right) + X_{i,n=10}^{j} * rop_{x=4} + X_{i,n=11}^{j} * rop_{x=5} + X_{i,n=1}^{j-1} * \psi_{i}^{j-1} + X_{i-1,n=12}^{j} * \varphi_{i-1}$$

TABLES (Full Model)

Table S1: Model Parameters, Sampling Distributions, and Sources. PLWH: people with HIV. MSM: men who have sex with men. PWID: people who inject drugs. SVR: sustained viral response. HCC: hepatocellular carcinoma.

Notation	Parameter Description	Unit	Mean Sampled Value	Sampling Distribution	Source
c	Sustained viral response (SVR) rate among HIV- individuals	annual rate	0.64 (<2013) 0.95 (≥2013)	Uniform (<2013) [0.59, 0.69] Uniform (≥2013) [0.90, 1.00]	1 2,3
c_{HIV}	Sustained viral response (SVR) rate among HIV+ individuals	annual rate	0.38 (<2013) 0.95 (≥2013)	Uniform (<2013) [0.35, 0.42] Uniform (≥2013) [0.90, 1.00]	4 2,3
p	Proportion of HIV- individuals that spontaneously clear HCV infection	-	0.26	Uniform [0.22, 0.29]	5
p_{HIV}	Proportion of HIV+ individuals that spontaneously clear HCV infection	-	0.15	Uniform [0.12, 0.18]	6
RR_{HIV_tr}	Relative risk of HCV transmission for HIV+ persons compared to HIV-	-	2.56	Lognormal [95% CI 1.5, 4.43]	7
RR_{HIV_s}	Relative HCV susceptibility for HIV+ persons compared to HIV-	-	1.9	Lognormal [95% CI 1.3, 2.7]	8
rop_1	TP^{++} of disease progression from mild (X_7) to moderate liver disease (X_8)	annual transition probability	0.025	Beta [38.086, 1485.3516]	9
rop_2	TP^{++} of disease progression from moderate liver disease (X_8) to compensated cirrhosis (X_9)	annual transition probability	0.037	Beta [26.905, 700.2582]	9
rop_3	TP^{++} of disease progression from compensated cirrhosis (X_9) to decompensated cirrhosis (X_{10})	annual transition probability	0.039	Beta [14.617, 260.1732]	9
rop_4	TP^{++} of disease progression from compensated cirrhosis (X_9) to HCC (X_{11})	annual transition probability	0.014	Beta [1.9326, 136.1074]	9
rop_5	TP^{++} of disease progression from decompensated cirrhosis (X_{10}) to HCC (X_{11})	annual transition probability	0.030	Beta [6.5256, 210.9945]	9
HR_{dc}	Hazard ratio of progression to decompensated cirrhosis after SVR compared to no SVR	ratio	0.07	Lognormal [95% CI 0.03, 0.20]	10
HR_{hcc}	Hazard ratio of progression to HCC after SVR compared to no SVR	ratio	0.23	Lognormal [95% CI 0.16, 0.35]	11
ν_1	Progression from decompensated cirrhosis (X_{10}) or susceptible decompensated cirrhosis (X_5) to death	annual transition probability	0.13	Beta [147.03, 983.97]	9
ν_2	Progression from HCC (X_{11}) or susceptible HCC (X_6) to death	annual transition probability	0.43	Beta [117.1, 155.23]	9

δ	IDU-related overdose mortality rate among PWID	annual rate	0.006	-	[12]
ε	IDU-related overdose mortality rate linear increase factor (2017-2020)	-	0 (<2017)<br>0.00106875 (2017-2021)	-	[13]
ψ	Rate of transition for PWID to ex-PWID, where ψ = 1/(IDU duration in years)	annual rate	0.049	Uniform [9, 32] from duration of IDU (years)	[14]
$\mu_{i=1}$	Background mortality rate for age stage 18-39	annual rate	0.001209	probability of dying between ages 28-29	[15]
$\mu_{i=2}$	Background mortality rate for age stage 40-54	annual rate	0.003091	probability of dying between ages 47-48	[15]
$\mu_{i=3}$	Background mortality rate for age stage 55-74	annual rate	0.011915	probability of dying between ages 64-65	[15]
$\mu_{i=4}$	Background mortality rate for age stage 75+	annual rate	0.081301	1/expectation of life at age 75-76	[15]
$tUCSD$	Annual proportion of HIV+ individuals treated from 2018 to 2021 at UCSD Owen Clinic	annual proportion	0.537	-	[16]
$trt_{i,x}^{j=4,8}$	Annual proportion of Non/Ex-PWID without HIV treated by disease stage	annual proportion	0.00 (<1996)<br>0.05 (≥1996)	-	[17,18]
$trt_{i,x}^{j=1,3,7}$	Annual proportion of MSM & PWID without HIV treated by disease stage	annual proportion	0.00 (< 2023)	-	[14]

[++]Annual transition probability was converted to annual transition rates in the model

Table S2: Calibrated parameters. PLWH: people with HIV. MSM: men who have sex with men. PWID: people who inject drugs.

Notation	Parameter Description	Unit	Mean Value	Obtained through calibration to
m	Degree of assortative mixing among HIV+ and non-HIV+ MSM	proportion	0.8122	Submodel
$beta_{MSM}$	Per capita number of effective contacts per unit time for MSM	annual rate	0.0470	Submodel
$beta_{PWID}$	Per capita number of effective contacts per unit time for PWID	annual rate	0.0892	Submodel
trt_1	Annual proportion of HIV+ individuals treated from 1996 to 2010 (countywide)	annual proportion	0.2074	Submodel
$trt_{i,x}^{j=2,5,6,9,10}$	Annual proportion of HIV+ individuals treated from 2015 to 2017 (countywide)	annual proportion	0.2703	Submodel
tNON	Annual proportion of HIV+ individuals treated from 2018 to 2021 at non-UCSD sites	annual proportion	0.6776	Submodel
RR_{PWID}	Relative risk of transmission among young PWID [age 18-39] compared to older PWID	annual rate	4.580	Submodel
RR_{PWID_time}	Year the elevated relative risk of transmission rate among young PWID starts	-	By model start (2015)	Submodel
ω	Reduction in HCV related mortality among chronically infected HCV individuals due to liver transplant	proportion	0.191	Full model
px	Proportion of HCV infected individuals who have previously attained SVR in 2015	proportion	0.285	Full model

Table S3: Initial conditions for full model initialized in 2015. MSM: men who have sex with men. PWID: people who inject drugs.

Population Sub-type	Point Estimate	Number HCV Seropositive	Data Source
MSM with HIV	16,902		[19-21]
MSM without HIV	71,396		[19-21]
Total MSM		6,229	[19-21]
Total PWID male	21,995		[19-22]
Total PWID female	8,135		[19,20,22,23]
Total PWID		8,446	[19-23]
Total Non-PWID male	1,200,515	20,807	[19]
Total Non-PWID female	1,298,993	11,438	[19]
Non-PWID male (18-39)	550,514	0	[19]
Non-PWID female (18-39)	530,782	0	[19]
Non-PWID male (40-54)	283,080	6,053	[19]
Non-PWID female (40-54)	307,829	3,141	[19]
Non-PWID male (55-74)	290,355	13,769	[19]
Non-PWID female (55-74)	345,081	8,297	[19]
Non-PWID male (75+)	76,566	985	[19]
Non-PWID female (75+)	115,301	0	[19]

Table S4: HCV disease distribution for non-PWID at initial conditions for full model initialized in 2015. PWID: people who inject drugs. Calculated from the age stratified disease progression model (without transmission) using a closed cohort of individuals.

**Population Sub-type [Disease stage(s)]**	**Proportion of HCV-infected individuals by disease stage within each age group**
Non-PWID (18-54) Mild HCV	0.2030
Non-PWID (18-54) Moderate HCV	0.4900
Non-PWID (18-54) Compensated Cirrhosis	0.2638
Non-PWID (18-54) Decompensated Cirrhosis	0.0274
Non-PWID (18-54) Hepatocellular Carcinoma	0.0158
Non-PWID (55+) Mild HCV	0.0066
Non-PWID (55+) Moderate HCV	0.0886
Non-PWID (55+) Compensated Cirrhosis	0.7056
Non-PWID (55+) Decompensated Cirrhosis	0.1238
Non-PWID (55+) Hepatocellular Carcinoma	0.0754

MODEL CALIBRATION PROCESS

We use a two-step model calibration process as described in the main text. For our first model calibration step, we use a simplified submodel (Equations below) which represents only PWID and MSM (excluding ex/non-PWID). For this submodel, we calibrated to data on HCV seroprevalence in 2015 among MSM (4.6% among all MSM, 16.5% among MSM with HIV) [19,24], HCV viremia prevalence among HCV seropositive PLWH of 42.1% (2010), 18.5% (2018) and 8.5% (2021) [16], the number of PWID in 2007 [19,25], HCV seroprevalence of 46% and 36% among young PWID (aged 18-39) and older PWID (aged 40-74) in 2021 respectively [14], and primary HCV incidence rate among PWID of 17.14 per 100 person-years in 2021 [14]. Calibration was obtained by varying the following parameters: transmission rate among MSM, transmission rate among PWID, degree of assortative mixing among MSM by HIV status, annual treatment rates among HIV/HCV co-infected individuals (1996-2010, 2011-2017, and 2018-2021), the relative risk of transmission among young PWID (aged 18-39) and start year of increased risk among young PWID. Then, using the calibrated parameters from the submodel, the full model is then additionally calibrated to the number of total HCV related deaths in 2015 (290) and 2019 (320) [26] in order to identify the following parameters: HCV related death rate scaling factor due to liver transplant, and the ratio of split between susceptible & infected among HCV antibody positive Non-PWID upon model initialization in 2015.

SUBMODEL EQUATIONS

This submodel excludes ex/non-PWID and was used for model calibration to parameters which did not require these population groups. A description of all values is provided in the tables above.

For the below equations:

i = age stage (1-4), where,
i = 1: 18-39-year olds
i = 2: 40-54-year olds
i = 3: 55-74-year olds
i = 4: 75+ year olds

j = population sub-type (1-6), where,
j = 1: MSM

j = 2: MSM HIV+
j = 3: PWID Male
j = 4: PWID HIV+ Male
j = 5: PWID Female
j = 6: PWID HIV+ Female

X_n denotes the infection/disease stages, where,
n = infection/disease stages, and
n = 1: Susceptible (X_1)
n = 2: Spontaneous Clearance from no/mild liver disease (X_2)
n = 3: SVR from no/mild liver disease (X_3)
n = 4: Susceptible Moderate Liver Disease (X_4)
n = 5: Susceptible Compensated Cirrhosis (X_5)
n = 6: Susceptible Decompensated Cirrhosis (X_6)
n = 7: Susceptible Hepatocellular Carcinoma (X_7)
n = 8: No/Mild Liver Disease (X_8)
n = 9: Moderate Liver Disease (X_9)
n = 10: Compensated Cirrhosis (X_{10})
n = 11: Decompensated Cirrhosis (X_{11})
n = 12: Hepatocellular Carcinoma (X_{12})

φ_i = population ageing rate, where,
φ_1 = ageing rate from i = 1 to i = 2
φ_2 = ageing rate from i = 2 to i = 3
φ_3 = ageing rate from i = 3 to i = 4
$\varphi_0 = \varphi_4 = 0$

μ_i = background mortality rate, where the rate is dependent on age

δ^j = Overdose mortality rate, where $\delta^{j=1,2,4,6,8,10} = 0$

$$\delta^{j=3,5,7,9} = \delta \; if \; t \leq 2017$$
$$\delta^{j=3,5,7,9} = \delta * \varepsilon \; if \; t = 2018$$
$$\delta^{j=3,5,7,9} = \delta * 2\varepsilon \; if \; t = 2019$$
$$\delta^{j=3,5,7,9} = \delta * 3\varepsilon \; if \; t = 2020$$
$$\delta^{j=3,5,7,9} = \delta * 4\varepsilon \; if \; t \geq 2021$$

ν_n = HCV-related death rate, where, $\nu_{n=1,2,3,4,5,8,9,10} = 0$

ω = Reduction in HCV related mortality among chronically infected HCV individuals due to liver transplant

$trt^j_{i,x}(t)$ = treatment proportion, where,

$trt^{j}_{i,x=1}(t)$ = proportion of individuals treated from no/mild liver disease ($X^{j}_{i,n=8}$)
$trt^{j}_{i,x=2}(t)$ = proportion of individuals treated from moderate liver disease ($X^{j}_{i,n=9}$)
$trt^{j}_{i,x=3}(t)$ = proportion of individuals treated from compensated cirrhosis ($X^{j}_{i,n=10}$)
$trt^{j}_{i,x=4}(t)$ = proportion of individuals treated from decompensated cirrhosis ($X^{j}_{i,n=11}$)
$trt^{j}_{i,x=5}(t)$ = proportion of individuals treated from HCC ($X^{j}_{i,n=12}$)

For 2017-2021 we simulated a weighted treatment rate between UCSD and non-UCSD clinics (with UCSD clinics providing care for an estimated κ=26% of people with HIV in San Diego), using historical treatment data from UCSD clinics and calibrating the non-UCSD rate to achieve the observed chronic prevalence declines among people with HIV, such that

$$trt^{j=2,5,6,9,10}_{i,x} = tUCSD * \kappa + tNON * (1 - \kappa) \quad if \;\; 2017 \;\leq t \; < 2021$$

HR_{DC} = Hazard ratio of progression to decompensated cirrhosis after SVR compared to no SVR
HR_{HCC} = Hazard ratio of progression to HCC after SVR compared to no SVR

rop_x = rate of disease progression, where,
$rop_{x=1}$ = rate of disease progression from ($X^{j}_{i,n=8}$) to ($X^{j}_{i,n=9}$)
$rop_{x=2}$ = rate of disease progression from ($X^{j}_{i,n=9}$) to ($X^{j}_{i,n=10}$)
$rop_{x=3}$ = rate of disease progression from ($X^{j}_{i,n=10}$) to ($X^{j}_{i,n=11}$)
$rop_{x=4}$ = rate of disease progression from ($X^{j}_{i,n=10}$) to ($X^{j}_{i,n=12}$)
$rop_{x=5}$ = rate of disease progression from ($X^{j}_{i,n=11}$) to ($X^{j}_{i,n=12}$)
$rop_{x=6}$ = rate of disease progression from ($X^{j}_{i,n=5}$) to ($X^{j}_{i,n=6}$) = $HR_{DC} * rop_{x=3}$
$rop_{x=7}$ = rate of disease progression from ($X^{j}_{i,n=5}$) to ($X^{j}_{i,n=7}$) = $HR_{DC} * rop_{x=4}$
$rop_{x=8}$ = rate of disease progression from ($X^{j}_{i,n=6}$) to ($X^{j}_{i,n=7}$) = $HR_{HCC} * rop_{x=5}$

p^j = proportion of individuals that spontaneously clear HCV infection which varies by HIV status:

$$\begin{cases} p^{j=1,3,4,7,8} = p \\ p^{j=2,5,6,9,10} = \text{pHIV} \end{cases}$$

$c^j\,(t)$ = proportion of individuals that attain sustained viral response (SVR) which varies by time and HIV status:

$$\begin{cases} c^{j=1,3,4,7,8} = c(t) \\ c^{j=2,5,6,9,10} = \text{cHIV(t)} \end{cases}$$

RR_{HIV_tr} = relative risk of transmission among HIV+ individuals
RR_{HIV_s} = relative risk of susceptibility among HIV+ individuals

As detailed in the main text, we simulate a time-varying risk of transmission among PWID in the youngest age category (age 18-39) compared to the older categories, with the year that this elevated risk starts found through model calibration:

$$\begin{cases} RR_{yPWID} = 1\ if\ t < YearRR \\ RR_{yPWID} = RR_{young}\ if\ t \geq YearRR \end{cases}$$

m = degree of assortative mixing among HIV+ and non-HIV+ MSM

ψ^j = rate of transition from PWID to ex-PWID, where,

$$\begin{cases} \psi_i^{j=0,1,2,4,6,8,10} = 0 \\ \psi_i^{j=3,5,7,9} = \psi \end{cases}$$

The entry rate is denoted by the term, α. We replaced all deaths with entries, excluding HCV and excess drug-related deaths from 2017 onwards (where the rate increases), such that the population is stable without HCV and without excess drug-related mortality from 2017 onwards.

Force of Infection Terms

People in HIV+ compartments having an additional relative risk term of transmissibility (RR_{HIV_tr}), and susceptibility (RR_{HIV_s}).

For MSM (*j* = 1, 2 and *i* = 1, 2, 3, 4)

$$\frac{I\_MSM\_HIV(t)}{N\_MSM\_HIV(t)} = \frac{\sum_{i=1}^{4} \sum_{n=8}^{12}(RR_{HIV\_tr} * X_{i,n}^{j=2})}{\sum_{i=1}^{4} \sum_{n=8}^{12}(RR_{HIV\_tr} * X_{i,n}^{j=2}) + \sum_{i=1}^{4} \sum_{n=1}^{7}(RR_{HIV_{tr}} * X_{i,n}^{j=2})}$$

$$\frac{I\_MSM\_HIVneg(t)}{N\_MSM\_HIVneg(t)} = \frac{\sum_{i=1}^{4} \sum_{n=8}^{12}(X_{i,n}^{j=1})}{\sum_{i=1}^{4} \sum_{n=8}^{12}(X_{i,n}^{j=1}) + \sum_{i=1}^{4} \sum_{n=1}^{7}(X_{i,n}^{j=1})}$$

$$\frac{I\_MSM(t)}{N\_MSM(t)} = \frac{\sum_{i=1}^{4} \sum_{n=8}^{12}(X_{i,n}^{j=1} + RR_{HIV\_tr} * X_{i,n}^{j=2})}{\sum_{i=1}^{4} \sum_{n=8}^{12}(X_{i,n}^{j=1} + RR_{HIV\_tr} * X_{i,n}^{j=2}) + \sum_{i=1}^{4} \sum_{n=1}^{7}(X_{i,n}^{j=1} + RR_{HIV_{tr}} * X_{i,n}^{j=2})}$$

Force of infection for MSM HIV-: $foi_i^{j=1} = \beta_{MSM} * \left(m * \left[\frac{I_{MSM_{HIVneg(t)}}}{N_{MSM_{HIVneg(t)}}}\right] + (1-m) * \left[\frac{I_{MSM(t)}}{N_{MSM(t)}}\right]\right)$

Force of infection for MSM HIV+: $foi_i^{j=2} = \beta_{MSM} * RR_{HIV_s} * \left(m * \left[\frac{I_{MSM_{HIV(t)}}}{N_{MSM_{HIV(t)}}}\right] + (1-m) * \left[\frac{I_{MSM(t)}}{N_{MSM(t)}}\right]\right)$

For PWID (*j* =3, 4, 5, 6 and *i* = 1, 2, 3, 4)

$$\frac{I\_PWID(t)}{N\_PWID(t)} = \frac{\sum_{n=8}^{12}(X_{i,n}^{j=3} + X_{i,n}^{j=5} + RR_{HIV\_tr} * (X_{i,n}^{j=4} + X_{i,n}^{j=6}))}{\sum_{n=8}^{12}(X_{i,n}^{j=3} + X_{i,n}^{j=5} + RR_{HIV\_tr} * (X_{i,n}^{j=4} + X_{i,n}^{j=6})) + \sum_{n=1}^{7}(X_{i,n}^{j=3} + X_{i,n}^{j=5} + RR_{HIV\_tr} * (X_{i,n}^{j=4} + X_{i,n}^{j=6})}$$

Force of infection for young PWID Male HIV-: $foi_{i=1}^{j=3} = \beta_{PWID\_m} * RR_{yPWID}(t) * \left[\frac{I\_PWID(t)}{N\_PWID(t)}\right]$

Force of infection for young PWID Male HIV+: $foi_{i=1}^{j=4} = \beta_{PWID\_m} * RR_{yPWID}(t) * RR_{HIV_s} * \left[\frac{I\_PWID(t)}{N\_PWID(t)}\right]$

Force of infection for young PWID Female HIV-: $foi_{i=1}^{j=5} = \beta_{PWID\_f} * RR_{yPWID}(t) * \left[\frac{I\_PWID(t)}{N\_PWID(t)}\right]$

Force of infection for young PWID Female HIV+: $foi_{i=1}^{j=6} = \beta_{PWID\_f} * RR_{yPWID}(t) * RR_{HIV_s} * \left[\frac{I\_PWID(t)}{N\_PWID(t)}\right]$

Force of infection for older PWID Male HIV-: $foi_{i=2,3,4}^{j=3} = \beta_{PWID\_m} * \left[\frac{I\_PWID(t)}{N\_PWID(t)}\right]$

Force of infection for older PWID Male HIV+: $foi_{i=2,3,4}^{j=4} = \beta_{PWID\_m} * RR_{HIV_s} * \left[\frac{I\_PWID(t)}{N\_PWID(t)}\right]$

Force of infection for older PWID Female HIV-: $foi_{i=2,3,4}^{j=5} = \beta_{PWID\_f} * \left[\frac{I\_PWID(t)}{N\_PWID(t)}\right]$

Force of infection for older PWID Female HIV+: $foi_{i=2,3,4}^{j=6} = \beta_{PWID\_f} * RR_{HIV_s} * \left[\frac{I\_PWID(t)}{N\_PWID(t)}\right]$

Model Equations
The model equations are as follows

$$\frac{dX_{n=1}}{dt} = \alpha_{i=1}^{j} - X_{i,n=1}^{j} * \left(foi_i^j * p^j + foi_i^j * \left(1 - p^j\right) + \mu_i + \varphi_i + \psi_i^j + \delta^j \right) + X_{i-1,n=1}^{j} * \varphi_{i-1}$$

$$\frac{dX_{n=2}}{dt} = X_{i,n=1}^{j} * foi_i^j * p^j - X_{i,n=2}^{j} * \left(foi_i^j * \left(1 - p^j\right) + \mu_i + \varphi_i + \psi_i^j + \delta^j \right) + X_{i-1,n=2}^{j} * \varphi_{i-1}$$

$$\frac{dX_{n=3}}{dt} = -X_{i,n=3}^{j} * \left(foi_i^j * \left(1 - p^j\right) + \mu_i + \varphi_i + \psi_i^j + \delta^j \right) + X_{i,n=8}^{j} * trt_{i,x=1}^{j}(t) * c^j\ (t) + X_{i-1,n=3}^{j} * \varphi_{i-1}$$

$$\frac{dX_{n=4}}{dt} = -X_{i,n=4}^{j} * \left(foi_i^j * \left(1 - p^j\right) + \mu_i + \varphi_i + \psi_i^j + \delta^j \right) + X_{i,n=9}^{j} * trt_{i,x=2}^{j}(t) * c^j\ (t) + X_{i-1,n=4}^{j} * \varphi_{i-1}$$

$$\frac{dX_{n=5}}{dt} = -X_{i,n=5}^{j} * \left(foi_i^j * \left(1 - p^j\right) + rop_{x=6} + rop_{x=7} + \mu_i + \varphi_i + \psi_i^j + \delta^j \right) + X_{i,n=10}^{j} * trt_{i,x=3}^{j}(t) * c^j\ (t) + X_{i-1,n=5}^{j} * \varphi_{i-1}$$

$$\frac{dX_{n=6}}{dt} = -X_{i,n=6}^{j} * \left(foi_i^j * \left(1 - p^j\right) + rop_{x=8} + \nu_n + \mu_i + \varphi_i + \psi_i^j + \delta^j \right) + X_{i,n=5}^{j} * rop_{x=6} + X_{i,n=11}^{j} * trt_{i,x=4}^{j}(t) * c^j\ (t) + X_{i-1,n=6}^{j} * \varphi_{i-1}$$

$$\frac{dX_{n=7}}{dt} = -X_{i,n=7}^{j} * \left(foi_i^j * \left(1 - p^j\right) + \nu_n + \mu_i + \varphi_i + \psi_i^j + \delta^j \right) + X_{i,n=12}^{j} * trt_{i,x=5}^{j}(t) * c^j\ (t) + X_{i,n=5}^{j} * rop_{x=7} + X_{i,n=6}^{j} * rop_{x=8} + X_{i-1,n=7}^{j} * \varphi_{i-1}$$

$$\frac{dX_{n=8}}{dt} = foi_i^j * \left(1 - p^j\right) * \left(X_{i,n=1}^{j} + X_{i,n=2}^{j} + X_{i,n=3}^{j}\right) - X_{i,n=8}^{j} * \left(trt_{i,x=1}^{j}(t) * c^j\ (t) + rop_{x=1} + \mu_i + \varphi_i + \psi_i^j + \delta^j \right) + X_{i-1,n=8}^{j} * \varphi_{i-1}$$

$$\frac{dX_{n=9}}{dt} = X_{i,n=4}^{j} * foi_i^j * \left(1 - p^j\right) - X_{i,n=9}^{j} * \left(trt_{i,x=2}^{j}(t) * c^j\ (t) + rop_{x=2} + \mu_i + \varphi_i + \psi_i^j + \delta^j \right) + X_{i,n=8}^{j} * rop_{x=1} + X_{i-1,n=9}^{j} * \varphi_{i-1}$$

$$\frac{dX_{n=10}}{dt} = X_{i,n=5}^{j} * foi_i^j * \left(1 - p^j\right) - X_{i,n=10}^{j} * \left(trt_{i,x=3}^{j}(t) * c^j\ (t) + rop_{x=3} + rop_{x=4} + \mu_i + \varphi_i + \psi_i^j + \delta^j \right) + X_{i,n=9}^{j} * rop_{x=2} + X_{i-1,n=10}^{j} * \varphi_{i-1}$$

$$\frac{dX_{n=11}}{dt} = X_{i,n=6}^{j} * foi_i^j * \left(1 - p^j\right) - X_{i,n=11}^{j} * \left(trt_{i,x=4}^{j}(t) * c^j \ (t) + rop_{x=5} + \nu_n \ + \mu_i \ + \varphi_i + \psi_i^j + \delta^j \ \right) + X_{i,n=10}^{j} * rop_{x=3} + X_{i-1,n=11}^{j} * \varphi_{i-1}$$

$$\frac{dX_{n=12}}{dt} = X_{i,n=7}^{j} * foi_i^j * \left(1 - p^j\right) - X_{i,n=12}^{j} * \left(trt_{i,x=5}^{j}(t) * c^j \ (t) + \ \nu_n \ + \mu_i \ + \varphi_i + \psi_i^j + \delta^j \ \right) + X_{i,n=10}^{j} * rop_{x=4} + X_{i,n=11}^{j} * rop_{x=5} + X_{i-1,n=12}^{j} * \varphi_{i-1}$$

TABLES (Submodel)

Table S5: Calibrated parameters for submodel. PLWH: people with HIV. MSM: men who have sex with men. PWID: people who inject drugs.

Notation	Parameter Description	Unit	Mean Value
m	Degree of assortative mixing among HIV+ and non-HIV+ MSM	proportion	0.8122
$beta_{MSM}$	Per capita number of effective contacts per unit time for MSM	annual rate	0.0470
$beta_{PWID}$	Per capita number of effective contacts per unit time for PWID	annual rate	0.0892
trt_1	Annual proportion of HIV+ individuals treated from 1996 to 2010 (countywide)	annual proportion	0.2074
trt_2	Annual proportion of HIV+ individuals treated from 2011 to 2017 (countywide)	annual proportion	0.2703
trt_4	Annual proportion of HIV+ individuals treated from 2018 to 2021 at non-UCSD sites	annual proportion	0.6776
num_init	Number of PWID at model initialization in 1955	-	30,257
RR_{PWID}	Relative risk of transmission among young PWID [age 18-39] compared to older PWID	annual rate	4.580
RR_{PWID_time}	Year the elevated relative risk of transmission rate among young PWID starts	-	2006

FIGURES

Supplementary Figure S1: Model projections of HCV incidence rate (per 100 person-years) among PWID in San Diego County (2015-2030) Mean model projections (lines), with shading denoting the 95% uncertainty interval around the status-quo scenario. Scenarios shown are: (1) status-quo treatment (black solid line).

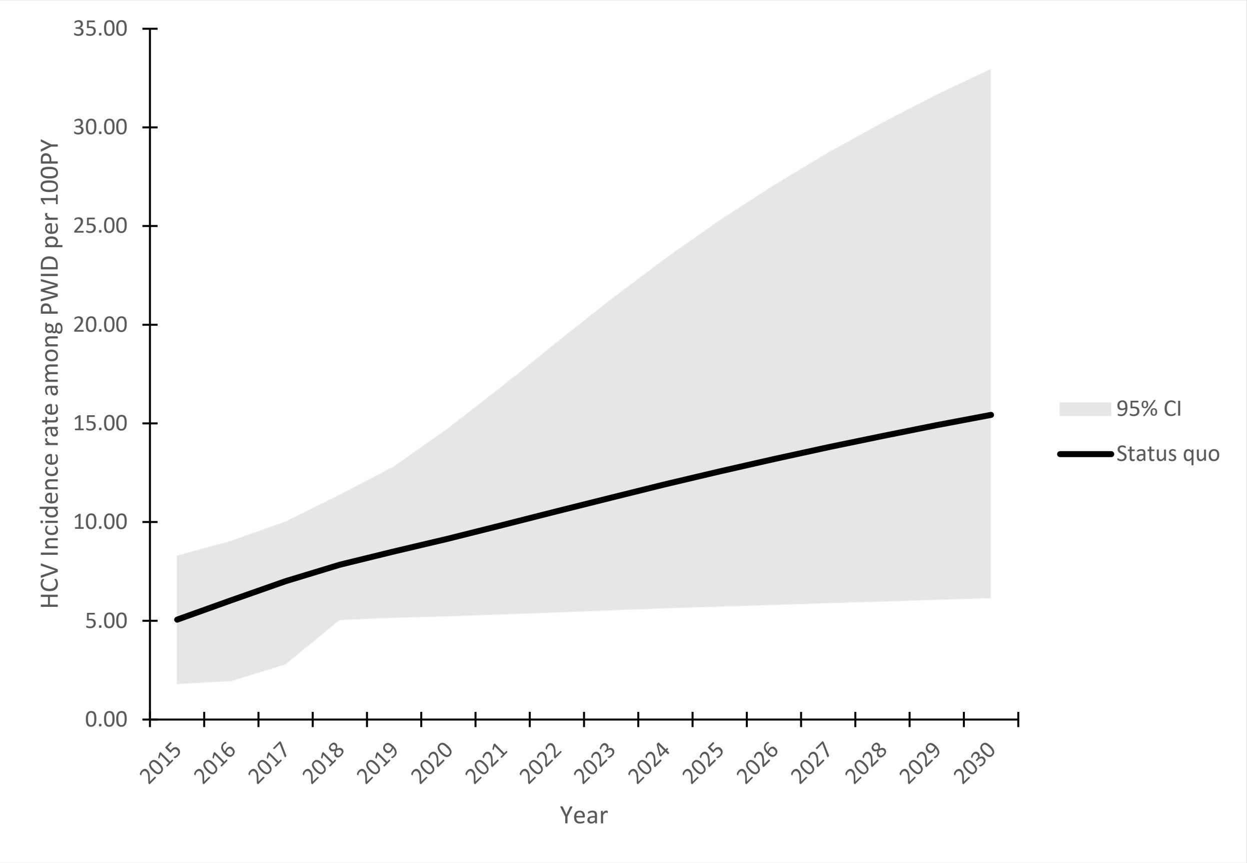

References

1. Borroni G, Andreoletti M, Casiraghi MA, et al. Effectiveness of pegylated interferon/ribavirin combination in 'real world' patients with chronic hepatitis C virus infection. *Aliment Pharmacol Ther*. May 2008;27(9):790-7. doi:10.1111/j.1365-2036.2008.03657.x
2. Scotto R, Buonomo AR, Moriello NS, et al. Real-World Efficacy and Safety of Pangenotypic Direct-Acting Antivirals Against Hepatitis C Virus Infection. *Rev Recent Clin Trials*. 2019;14(3):173-182. doi:10.2174/1574887114666190306154650
3. Hézode C. Treatment of hepatitis C: Results in real life. *Liver Int*. Feb 2018;38 Suppl 1:21-27. doi:10.1111/liv.13638
4. Davies A, Singh KP, Shubber Z, et al. Treatment outcomes of treatment-naïve Hepatitis C patients co-infected with HIV: a systematic review and meta-analysis of observational cohorts. *PLoS One*. 2013;8(2):e55373. doi:10.1371/journal.pone.0055373
5. Micallef JM, Kaldor JM, Dore GJ. Spontaneous viral clearance following acute hepatitis C infection: a systematic review of longitudinal studies. *J Viral Hepat*. Jan 2006;13(1):34-41. doi:10.1111/j.1365-2893.2005.00651.x
6. Thomson EC, Fleming VM, Main J, et al. Predicting spontaneous clearance of acute hepatitis C virus in a large cohort of HIV-1-infected men. *Gut*. 2011;60(6):837-845. doi:10.1136/gut.2010.217166
7. Benova L, Mohamoud YA, Calvert C, Abu-Raddad LJ. Vertical transmission of hepatitis C virus: systematic review and meta-analysis. *Clin Infect Dis*. Sep 15 2014;59(6):765-73. doi:10.1093/cid/ciu447
8. Frederick T, Burian P, Terrault N, et al. Factors associated with prevalent hepatitis C infection among HIV-infected women with no reported history of injection drug use: the Women's Interagency HIV Study (WIHS). *AIDS Patient Care STDS*. Nov 2009;23(11):915-23. doi:10.1089/apc.2009.0111
9. Shepherd J, Jones J, Hartwell D, Davidson P, Price A, Waugh N. Interferon alpha (pegylated and non-pegylated) and ribavirin for the treatment of mild chronic hepatitis C: a systematic review and economic evaluation. *Health Technol Assess*. Mar 2007;11(11):1-205, iii. doi:10.3310/hta11110
10. van der Meer AJ, Veldt BJ, Feld JJ, et al. Association between sustained virological response and all-cause mortality among patients with chronic hepatitis C and advanced hepatic fibrosis. *Jama*. Dec 26 2012;308(24):2584-93. doi:10.1001/jama.2012.144878
11. Morgan RL, Baack B, Smith BD, Yartel A, Pitasi M, Falck-Ytter Y. Eradication of hepatitis C virus infection and the development of hepatocellular carcinoma: a meta-analysis of observational studies. *Ann Intern Med*. Mar 5 2013;158(5 Pt 1):329-37. doi:10.7326/0003-4819-158-5-201303050-00005
12. Bradley H, Hall EW, Asher A, et al. Estimated Number of People Who Inject Drugs in the United States. *Clin Infect Dis*. Jan 6 2023;76(1):96-102. doi:10.1093/cid/ciac543
13. Branch SEaIS, Agency HaHS, Diego CoS. Opioid-Related Overdoses & Encounters In San Diego County, 2016-2020. Updated April 2023. https://www.sandiegocounty.gov/content/dam/sdc/hhsa/programs/phs/CHS/OD2A%202016-2020%20Retrospective%20Analysis.pdf
14. Strathdee SA. Data from: La Frontera. 2022.
15. Arias E, Xu J. *United States Life Tables, 2018*. Vol. 69. 2020. *National Vital Statistics Reports*.

16. Cachay E TF, Hill L, Ballard C, Aquino A, Qin H, Jain S, Martin S, Mathews WmC. Progress and real-life challenges for HCV elimination in People living with HIV. presented at: Conferences on Retrovirus and Opportunistic Infections; March 8-11 2020; Boston, USA.
17. Thomas DL. State of the Hepatitis C Virus Care Cascade. *Clin Liver Dis (Hoboken)*. Jul 2020;16(1):8-11. doi:10.1002/cld.915
18. Facente SN, Grebe E, Burk K, et al. Estimated hepatitis C prevalence and key population sizes in San Francisco: A foundation for elimination. *PLoS One*. 2018;13(4):e0195575. doi:10.1371/journal.pone.0195575
19. Wynn A, Tweeten S, McDonald E, et al. The estimated hepatitis C seroprevalence and key population sizes in San Diego in 2018. *PLoS One*. 2021;16(6):e0251635. doi:10.1371/journal.pone.0251635
20. Local Data: San Diego County, CA. AIDSVu. Updated April 14. Accessed May 21, 2021. https://aidsvu.org/local-data/united-states/west/california/san-diego-county/
21. *HIV Disease Among Adult/Adolescent Males, San Diego County, 2017*. 2017:22. https://www.sandiegocounty.gov/content/dam/sdc/hhsa/programs/phs/documents/Adult_Adol_Male_Through_12312017_final.pdf
22. Robertson AM, Garfein RS, Wagner KD, et al. Evaluating the impact of Mexico's drug policy reforms on people who inject drugs in Tijuana, B.C., Mexico, and San Diego, CA, United States: a binational mixed methods research agenda. *Harm Reduct J*. Feb 12 2014;11:4. doi:10.1186/1477-7517-11-4
23. *HIV Disease Among Females in San Diego County, 2017*. 2017:26. https://www.sandiegocounty.gov/content/dam/sdc/hhsa/programs/phs/documents/Females_Through_12312017v04-11-19.pdf
24. Horyniak D, Wagner KD, Armenta RF, Cuevas-Mota J, Hendrickson E, Garfein RS. Cross-border injection drug use and HIV and hepatitis C virus seropositivity among people who inject drugs in San Diego, California. *Int J Drug Policy*. Sep 2017;47:9-17. doi:10.1016/j.drugpo.2017.06.006
25. Tempalski B, Pouget ER, Cleland CM, et al. Trends in the population prevalence of people who inject drugs in US metropolitan areas 1992-2007. *PLoS One*. 2013;8(6):e64789. doi:10.1371/journal.pone.0064789
26. Prevention CfDCa. National Vital Statistics System, Mortality 1999-2020 on CDC WONDER Online Database. Accessed July 1, 2022. http://wonder.cdc.gov/ucd-icd10.html
